# Image-derived input functions from dynamic ^15^O–water PET scans using penalised reconstruction

**DOI:** 10.1186/s40658-023-00535-w

**Published:** 2023-03-07

**Authors:** Peter Young, Lieuwe Appel, Andreas Tolf, Savvas Kosmidis, Joachim Burman, Anna Rieckmann, Michael Schöll, Mark Lubberink

**Affiliations:** 1 Department of Psychiatry and Neurochemistry, University of Gothenburg, Wallinsgatan 6, 41341 Mölndal, Gothenburg, Sweden; 2 Wallenberg Centre for Molecular and Translational Medicine, University of Gothenburg, Gothenburg, Sweden; 3 Nuclear Medicine and PET, Department of Surgical Sciences, Uppsala University, Uppsala, Sweden; 4 Department of Medical Sciences, Neurology, Uppsala University, Uppsala, Sweden; 5 Department of Radiation Sciences, Umeå University, Umeå, Sweden; 6 Munich Center for the Economic of Aging, Max Planck Institute for Social Law and Social Policy, Munich, Germany; 7 Dementia Research Centre, Queen Square Institute of Neurology, University College London, London, UK

**Keywords:** ^15^O–water PET, Cerebral blood flow, Image-derived input function, Partial volume correction, PET

## Abstract

**Background:**

Quantitative positron emission tomography (PET) scans of the brain typically require arterial blood sampling but this is complicated and logistically challenging. One solution to remove the need for arterial blood sampling is the use of image-derived input functions (IDIFs). Obtaining accurate IDIFs, however, has proved to be challenging, mainly due to the limited resolution of PET. Here, we employ penalised reconstruction alongside iterative thresholding methods and simple partial volume correction methods to produce IDIFs from a single PET scan, and subsequently, compare these to blood-sampled input curves (BSIFs) as ground truth. Retrospectively we used data from sixteen subjects with two dynamic ^15^O-labelled water PET scans and continuous arterial blood sampling: one baseline scan and another post-administration of acetazolamide.

**Results:**

IDIFs and BSIFs agreed well in terms of the area under the curve of input curves when comparing peaks, tails and peak-to-tail ratios with R^2^ values of 0.95, 0.70 and 0.76, respectively. Grey matter cerebral blood flow (CBF) values showed good agreement with an average difference between the BSIF and IDIF CBF values of 2% ± and a coefficient of variation (CoV) of 7.3%.

**Conclusion:**

Our results show promising results that a robust IDIF can be produced for dynamic ^15^O–water PET scans using only the dynamic PET scan images with no need for a corresponding MRI or complex analytical techniques and thereby making routine clinical use of quantitative CBF measurements with ^15^O–water feasible.

**Supplementary Information:**

The online version contains supplementary material available at 10.1186/s40658-023-00535-w.

## Introduction

Positron emission tomography (PET) is used to quantify the distribution of radioactively labelled tracers of interest in the human body. Quantification in PET studies typically requires an input function to determine the kinetics of the radiotracers within the various tissues [1]. In many research studies, this input function is obtained through arterial cannulation and collection of blood samples either through manual collection or through using specialist equipment to obtain a blood-sampled input function (BSIF) [2]. Whilst arterial blood sampling is assumed to be the gold standard in obtaining an input function, it is not without practical difficulties. Arterial blood sampling is burdensome, subject to errors, and carries a small but not insignificant risk of adverse effects [3–6]; therefore, alternative solutions have the potential to be more accurate as well as to present lower risks to the participant during the procedure.

In this study, we have used ^15^O–water to measure cerebral blood flow (CBF), ^15^O–water is freely diffusible, metabolically inert, has an extraction fraction close to one and is hence considered the gold standard for non-invasive CBF quantification. ^15^O–water quantification traditionally requires an arterial input function; hence, the use of ^15^O–water for CBF measurements has not been implemented in clinical routine except in a small number of research centres due to the previously discussed problems. However, it is known that changes in brain perfusion are involved in many neurological and neurodegenerative disorders [7], and as such practical access to accurate, non-invasive, quantitative imaging of CBF may be beneficial to the future understanding of diseases where CBF measures provide insight.

One common approach to removing the requirement for a BSIF is a population-based input function (PBIF) which is based on the individual scaling of a radiopharmaceutical and population-specific input curve [8, 9]. Whilst few studies have demonstrated certain utility to this method, it has not been widely adopted. This is at least in part due to difficulties with non-matching injection protocols as well as the need for available data in a population that matches the study population being investigated.

Image-derived input functions (IDIFs) are another approach to determining the input curve by using a blood pool found within the PET image field-of-view (FoV). For neurological PET, this typically only refers to the carotid arteries as no other large blood pools are present in the FoV. One method to calculate an IDIF involves the delineation of the carotids using a high-resolution magnetic resonance image (MRI) and subsequently co-registering this to the PET image, using the MRI-derived carotid mask for measuring the input function on the PET image. This method produces good agreement with gold standard techniques; however, this method requires an MRI to be acquired in addition to the PET scan as well as additional analysis pipelines to segment the MRI and co-register it to the PET scan [10–12] which may not always be feasible for particular cohorts [13–15] or in certain clinical settings. IDIFs do have some disadvantages, for example, metabolite corrections are still required when using this method. Additionally, when using IDIFs in neurological PET the size of the carotid arteries is very close to the resolution of a PET scanner, leading to large partial volume effects (PVEs).

State-of-the-art PET scanners have produced IDIFs using PET images alone by delineating the carotid arteries using the high resolution afforded by these systems. This shows that accurate IDIFs can be derived from the PET images alone [16]. However, these ultra-high-resolution systems are not yet widely available and compensatory techniques for scanners with lower resolutions are required until these higher resolution systems are widely available [17]. Other methods have attempted to estimate the blood time–activity curve directly on PET scans using artificial intelligence (AI) [18]. Most of these methods, although resulting in excellent within-subject correlations across regions, exhibit a bias between BSIF and IDIF-based CBF values that varies considerably across subjects. Furthermore, especially AI-based methods suffer from the uncertainty of whether they can be used on other patient groups or perfusion states, than the ones they were trained on. Zanotti-Fregonara and colleagues [19] recently provided a thorough overview of methods published up until that point and concluded that although IDIFs were an attractive method for obtaining an input curve, there were few tracers to which IDIF methods could be applied without considerable implementation challenges.

As previously discussed, one major challenge in the accurate quantification of activity concentration in carotid arteries is PVEs, a phenomenon of either overestimating or underestimating the activity in a PET image or volume of interest (VOI) due to the limited resolution of the scanner [20, 21]. These factors cause blurring in subsequent images unless accounted for through partial volume correction (PVC) techniques [22–24]. Typically, in brain imaging, the PVE is defined as a combination of the spill-out of radioactivity from areas of high activity into surrounding tissue and the spill-in of radioactivity from areas of high activity into the VOI. Carotid arteries are small in diameter, typically around 5 mm [25], and thus particularly sensitive to PVE. Accurate PVC requires both a high spatial resolution, a good knowledge of spatial resolution in the images and a correct segmentation of the carotid arteries.

Clinical PET imaging typically employs ordered subset expectation maximisation (OSEM) reconstruction algorithms, which is a type of maximised likelihood expectation maximisation (MLEM). MLEM or OSEM tends to suffer from high noise when they are allowed to run to full convergence due to the sparse count statistics present in a typical PET data set. To reduce the noise, the algorithms will be stopped after a certain number of iterations. However, this leads to reductions in the accuracy of the data, with potential small object distortion and underestimation of activity concentrations in small objects. Block sequential regularised expectation maximisation (BSREM) [26], which has recently become commercially available on certain clinical PET systems, is a Bayesian penalised likelihood reconstruction algorithm which includes an additional noise penalization term into the objective function. This addition allows the algorithm to reach full convergence without the problems discussed previously introduced by OSEM. Increased accuracy and improved ability to resolve small objects are key advantages required to resolve the carotid arteries in PET images.

Historically, simpler methods for the determination of the carotid mask directly have been less successful due to limited scanner resolution and less advanced reconstruction algorithms [10, 13–15, 27]. With higher noise in images leading to difficulties in thresholding and relatively poor resolution complicating PVC, it has traditionally been difficult to create IDIFs using the PET image alone. However, the introduction of BSREM and resolution recovery methods leads to reduced noise and higher signal-to-noise ratio (SNR), as well as higher spatial resolution, which can create the possibility of using these techniques with accurate quantitative outcomes [28]. Hence, the present work aimed to evaluate a thresholding method on penalised likelihood images combined with PVC for the estimation of ^15^O–water IDIFs.

## Materials and methods

### Scan procedure

Anonymised data from sixteen subjects enrolled in a clinical study were included in the present work. Eight subjects were healthy controls (mean age 38.6), and eight subjects were diagnosed with multiple sclerosis (MS) (mean age 43.1). MS patients' data were included due to their scans being available from a clinical parent study involving the investigation of the effect of acetazolamide on CBF in MS patients. ^15^O–water PET data with accompanying blood measures are rare and so all data from this other study were included to maximise the amount of data available. All subjects gave their informed consent before inclusion, and the study was approved by the medical ethics review board in Uppsala (2014/453).

Each subject underwent two dynamic PET scans both starting simultaneously with a controlled bolus injection of 5 MBq/kg of ^15^O–water (10 ml at 1 ml/s followed by a 30-ml saline flush at 2 ml/s) on a GE Discovery MI PET/CT scanner (GE Healthcare, Waukesha) [29]. Approximately 10 min before the second scan, the subjects were administered acetazolamide (9 mg/kg up to 1 g). The pharmacological manipulation was not of interest to the current study—all scans were included. The time between scan starts was approximately 20 min to allow for radioactive decay.

### Blood sampling

Blood sampling at 3 ml/min was performed using an automated blood sampling system. This system measures activity in blood via a 6-cm-thick bismuth germanate crystal connected to a photomultiplier tube and multichannel analyser (MCA). The minimal readout time of the MCA is 1 s. (Veenstra-Comercer, Joure, The Netherlands) [30]. Two additional 2 ml blood samples were taken at 5 and 10 min post-injection and measured in cross-calibrated well counters for calibration of the individual online blood curves. Arterial input curves were corrected for the delay and dispersion [31] and resampled to the same frame times as the PET data. Dispersion correction was done using a fixed constant of 13 s, based on a measured dispersion of 8 s in the sampling system (data not shown) and an additional 5 s dispersion in the body [32].

### Image reconstruction

The ^15^O–water PET scans were reconstructed into 26 frames of variable duration (8 × 5 s, 4 × 10 s, 1 × 15 s, 4 × 20 s, 2 × 30 s, 7 × 60 s) onto a 256 × 256x71 matrix of 0.98 × 0.98x2.79 mm^3^ voxels using time-of-flight BSREM with a noise penalisation factor (β-value) of 300 (Q.Clear, GE Healthcare) [26] including resolution recovery as well as applying OSEM with 3 iterations, 16 subsets and a 5-mm Gaussian filter. All necessary corrections for quantitative images were applied according to the software supplied by the manufacturer.

### Carotid definition and PVC

**Fig. 1:**
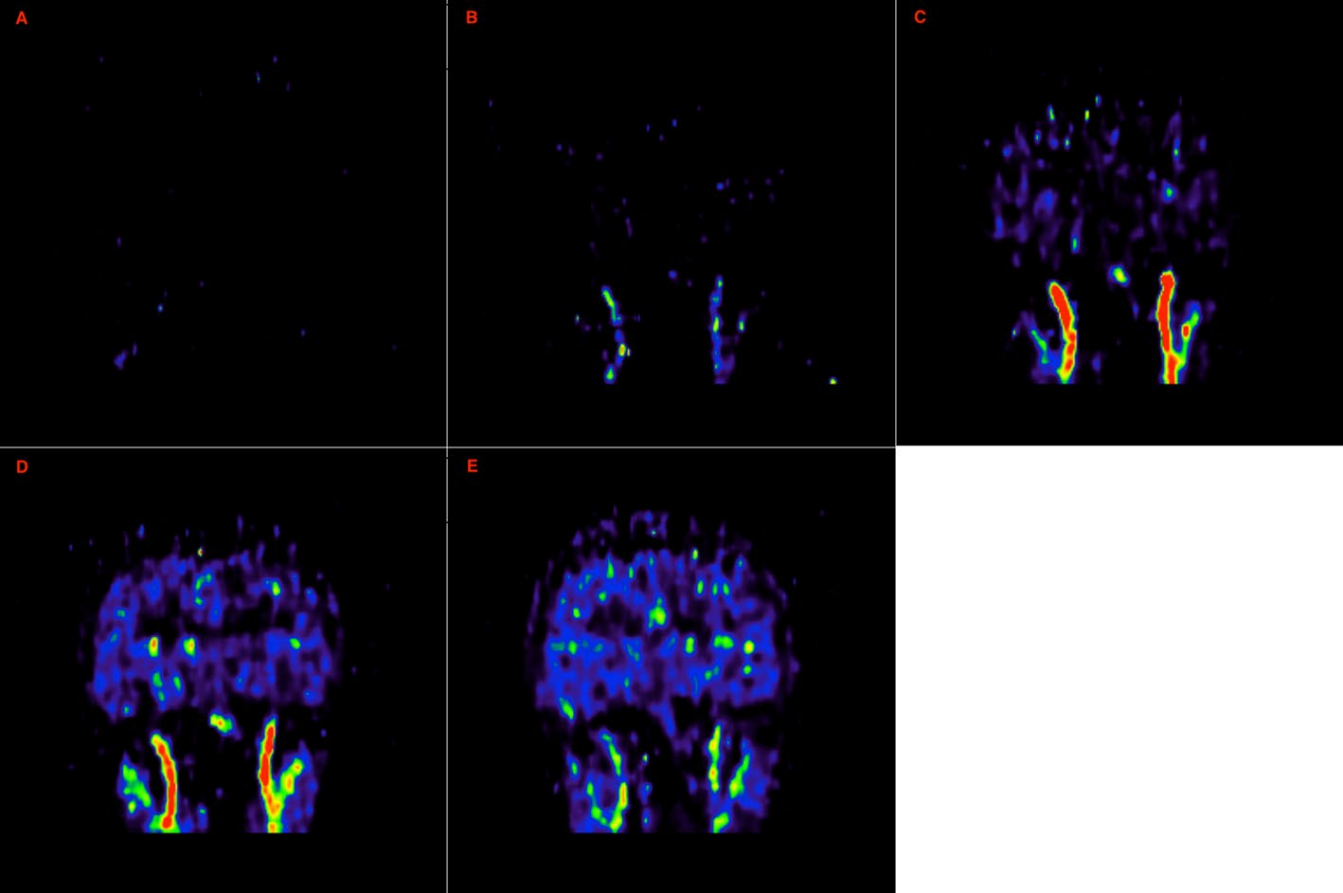
Frames 3 (**A**), 4 (**B**), 5 (**C**), 6 (**D**) and 7 (**E**) of an example subject whereby **A** would be excluded due to no presence of tracer in the carotids. **B**–**D** are included in the averaged image due to presence of signal in the carotid and **E** would be excluded due to the carotids no longer being discernible in the image. As **C** has the carotids best defined, this image would be used to measure the *x*, *y* and *z* coordinates to draw an VOI around the carotids to define a carotid mask

**Fig. 2:**
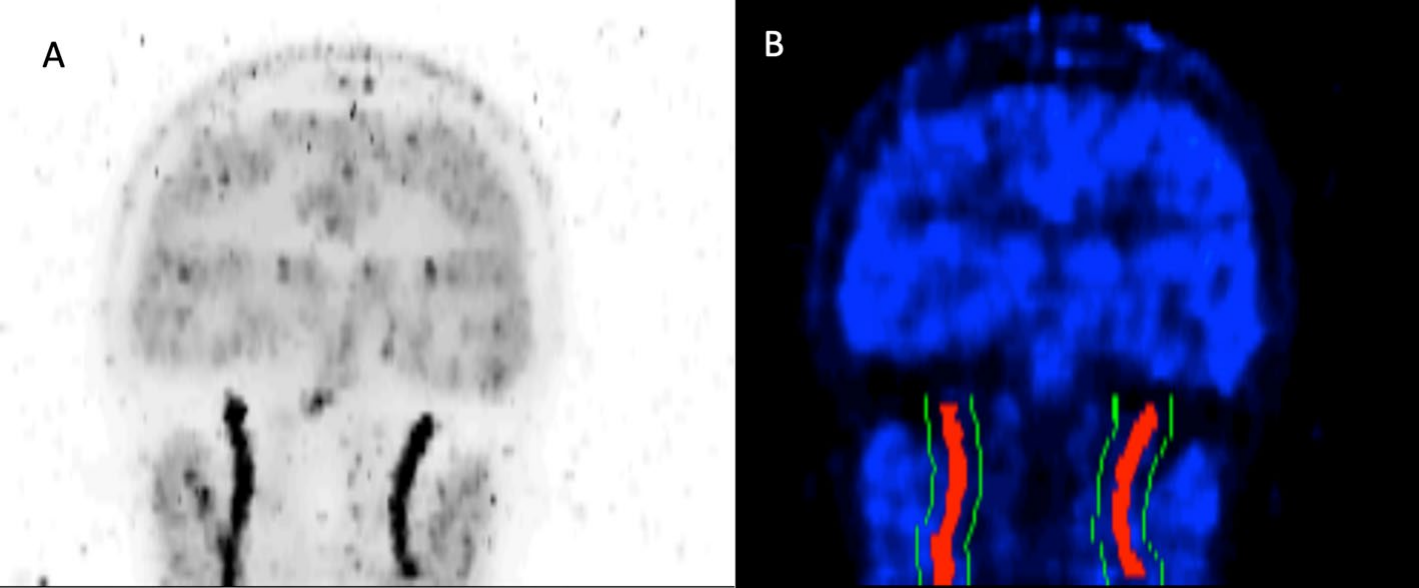
Example subject maximum intensity projection over the entire dynamic scan (**A**) and binary carotid mask (red) and surrounding tissue mask (green) overlayed onto a single frame of a dynamic ^15^O–water PET scan (**B**)

During the early frames of the ^15^O–water scans, the carotid arteries can be isolated by intensity thresholding due to the presence of the tracer in the carotids before it enters the brain tissue. For this step, the images were initially masked to the neck region of the PET image to improve the isolation of the carotids. This was performed by defining a volume of interest (VOI) consisting of the bottom third of the image FoV to exclude most of the brain. The early frames of the PET image were averaged and subsequently thresholded at a percentage of the median of the maximum pixel values in each slice across these early frames. These early frames were identified by manual inspection of the individual frames at the beginning of the scan up to frame 6 (corresponding to the first 30 s) to identify when the tracer had entered the carotid arteries and so are well defined before being more dissipated in the vascular system. Early frames were deemed acceptable if the entire internal carotids contained enough signal to be visually distinguishable from background but with little to no signal still in the brain. Each subject would also have a unique VOI drawn around the carotid arteries, with the x, y and z coordinates measured and applied on the image to maximise the isolation of the carotids for further assessment and minimise noise. Figure 1 gives a demonstration of the method by which the frame and coordinate choices were made. An average image of the chosen frames would then be created. The median value of the maximum pixel values in each slice was created by dividing the averaged image into axial slices and tabulating the maximum pixel intensity in each slice. This resultant thresholded image was then binarised to produce a carotid mask which was overlaid on the full time series of the PET scan to extract a time–activity curve (TAC). An example of this can be seen in Fig. 2.

PVE was estimated as follows: To determine underestimation due to spill-out, the carotid mask was smoothed with a Gaussian filter and then multiplied with the binary mask. Dividing the sum of all values in the smoothed mask with the sum of all the values (i.e. the number of nonzero voxels) in the binary mask gives the spill-out error (α). To account for spill-in, the binarised carotid mask was inverted, smoothed with the same Gaussian filter, multiplied with the carotid mask and division of the sum of all values in the resulting image with the number of nonzero voxels in the carotid mask gives the spill-in error (β). To estimate the surrounding tissue TAC, an 8-voxel dilation of the carotid mask was subtracted from a 10-voxel dilation of the carotid mask. These dilations were experimentally determined to be outside of any spillover effects from the carotids whilst remaining within the neck region for all patients to allow measurement of the tissue TAC. This created a sleeve around the carotids, thought to be representative of the tissue surrounding the carotids but not suffering from spillover from the carotids. The PVE-corrected blood curve *C*_B_(*t*) was then calculated as follows:

$$C_{{\text{B}}} \left( t \right) = \frac{1}{\alpha }\left( {C_{{\text{B}}}^{{{\text{PET}}}} \left( t \right) - \beta \times C_{{\text{T}}} \left( t \right)} \right)$$

Here, *C*_B_^PET^(*t*) is the uncorrected TAC of the carotid mask, and *C*_T_(*t*) is the surrounding tissue TAC.

### Optimisation of Gaussian kernel and mask threshold

The optimal values of the threshold used to create the carotid artery mask and the Gaussian kernel used for PVC were determined based on the area under the curve (AUC) of IDIFs compared to BSIF as well as the resulting whole-brain grey matter CBF values (see next section). AUC of peaks (0–60 s) and tails (60 s–5 min) of IDIFs, as well as peak-to-tail ratio, was calculated for a range of threshold values (38–46%) and Gaussian kernel widths (2.0–2.4 mm) around the theoretically expected optimal values. These were based on a threshold value of 41% resulting in a correct volume provided the object is homogeneous and sufficiently large compared to the spatial resolution [33] and reconstruction of the NEMA NU2:2012 spatial resolution measurement resulting in a spatial resolution of 2.6 mm (data not shown). Since this last measurement is done using 1.1-mm inner diameter glass capillaries, the true resolution is expected to be somewhat smaller than 2.6 mm. The mean relative bias in AUC for the peaks, tails, peak-to-tail ratios as well as grey matter (GM) CBF values across all subjects and scans was calculated to determine the optimal threshold and kernel settings. This relative bias was calculated by taking the average of the percentage differences between IDIFs and BSIFs. Additionally, PET images were reconstructed and placed through the same processing pipeline to identify if similar results can be found for OSEM reconstructions with different kernels as compared to the BSREM reconstructions.

Additional validation was performed using a phantom study. A 6-mm-diameter ^68^ Ga-filled (2.1 MBq/ml) tube was submerged in water in a 20-cm-diameter cylindrical phantom and a 10-min PET acquisition was performed. Images were reconstructed using the same settings as the patient images. Masking and PVC were applied to the image slices where the tube was approximately parallel to the scanner axis (ca. 5 cm length) as described above, again using 38–46% thresholds and 2.0–2.4-mm kernel widths, and the bias of the resulting radioactivity concentrations relative to the known concentration was calculated for each threshold and kernel value.

### Cerebral blood flow

CBF values and images were calculated using a basis function implementation of the single-tissue compartment model [34–36], as implemented in the Turku PET Centre library tools [37], using either the BSIF or IDIF as input function. Only the first 5 min of the acquisition were used. Regional cerebral blood flow (rCBF) values were extracted for all regions as defined using the Hammers atlas [38] after spatial normalisation to a common MNI (ICBM 152 [39, 40]) space using FSL [41, 42] (Additional file 1: Fig. S1).

### Statistics

The accuracy and precision of the IDIF-based CBF and *V*_d_ values compared to the BSIF-based values were assessed using Pearson’s correlation coefficient, orthogonal regression and Bland–Altman analysis. This was done across subjects using whole-brain GM CBF values, and within subjects using all CBF values for all regions included in the template.

### Automation

The method for isolating the carotids and building the carotid mask described above currently requires manual intervention for each subject with a choice of the frames to include as well as the selection of x, y and z coordinates for the VOI. An automated method was also employed which used region growing methods to create the carotid masks as well as using the same frames and coordinates for the VOI to isolate the carotids. The details of the automated method and results can be found in the Supplementary Material.

## Results

### Optimisation of Gaussian kernel and mask threshold

**Fig. 3:**
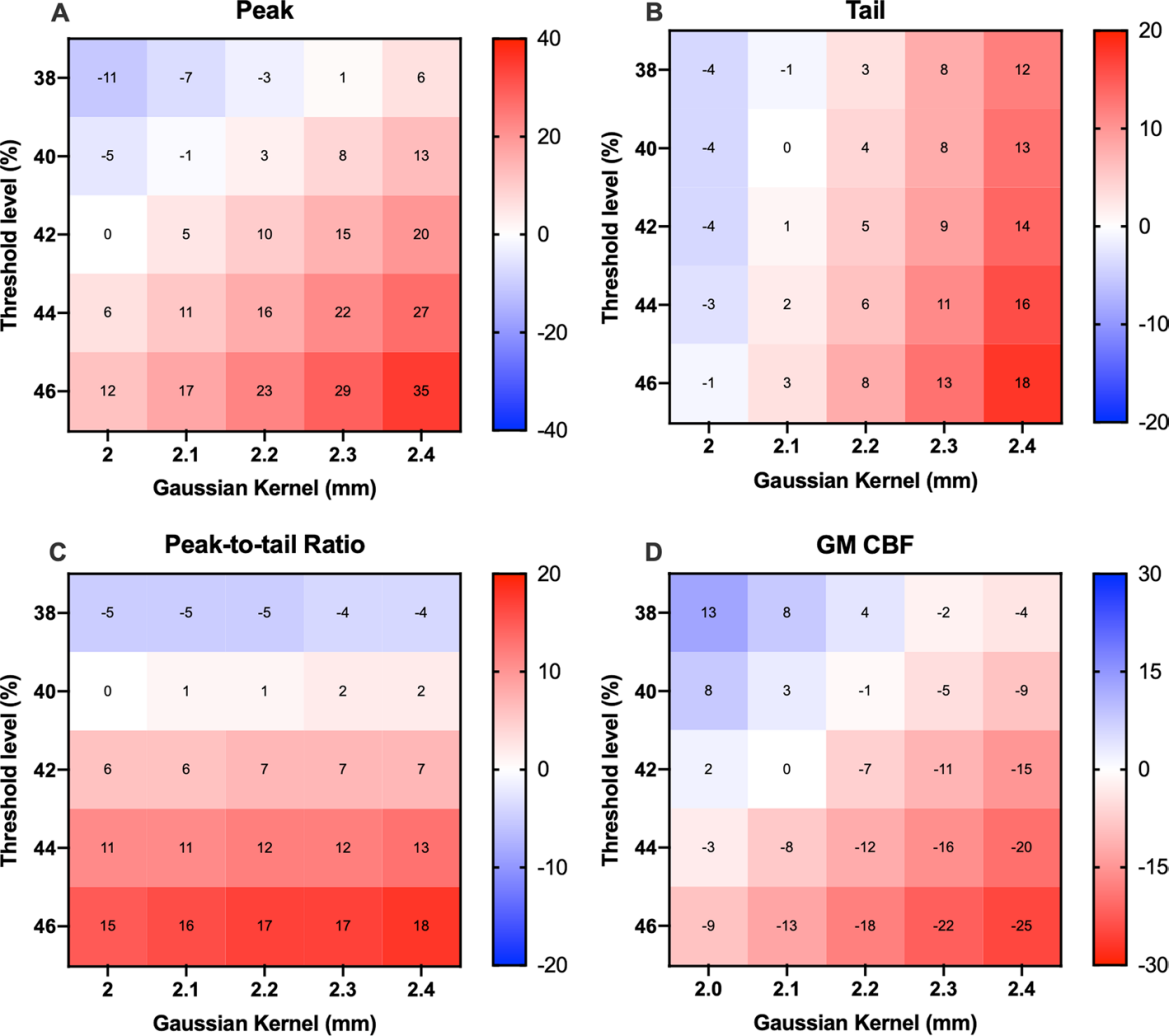
Mean percentage bias of AUC of peaks (**A**) and tails (**B**) and the bias of peak-to-tail ratios (**C**) and GM CBF (**D**) for a range of Gaussian kernels and threshold levels using BSREM reconstruction. *AUC* area under the curve, *GM* grey matter, *CBF* cerebral blood flow

Figure 3 shows the relative percentage bias in IDIF AUC as well as the whole GM CBF for each combination of thresholds and Gaussian kernels. A threshold value of 42% and a Gaussian kernel width of 2.0 mm resulted in the lowest peak AUC bias, whereas a 40% threshold and 2.1-mm kernel were optimal for tail AUC. Since bias for whole-brain GM CBF was lowest for a 42% threshold and a 2.1-mm kernel, this combination was evaluated further. This was confirmed in the phantom measurement, with the image-based radioactivity concentration after masking and PVC within 1% of the radioactivity measured using a dose calibrator for a 42% threshold and 2.1-mm kernel. Additional results using the automation method for optimisation of the Gaussian kernel and mask threshold can be found in the supplementary material. Additional file 1: Fig. S2 shows the same analysis performed on OSEM reconstruction images, with the lowest bias in peak, tails and CBF having little convergence. The OSEM results demonstrated much higher variance compared to the BSREM results and were not evaluated further.

### Input curves

**Fig. 4:**
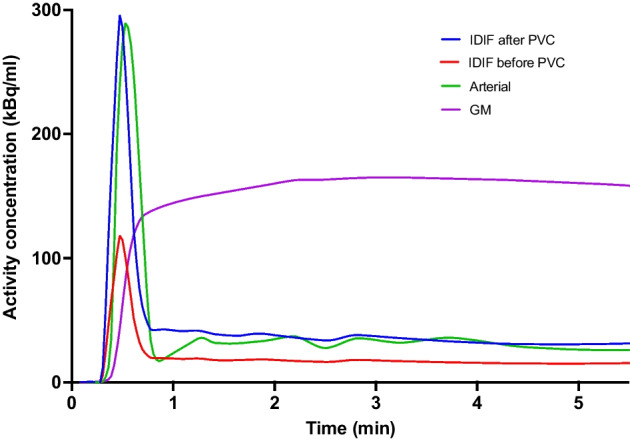
Input curves for representative subject: BSIF (green), IDIF before PVC (red), IDIF after PVC (blue) and GM activity concentration (purple). *BSIF* blood-sampled input function. *IDIF* Image-derived input function. *PVC* partial volume correction. *GM* Grey matter

**Fig. 5:**
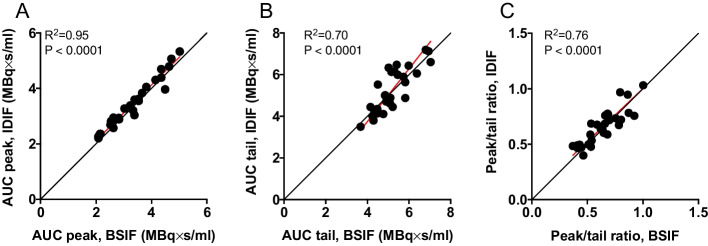
Scatter plots comparing peak (**A**) and tail (**B**) AUC of BSIF and IDIF, as well as peak-to-tail ratio (**C**). The black lines are lines of identity and the red lines are orthogonal regressions

Figure 4 shows a representative example of IDIFs both before and after PVC using a 42% threshold and 2.1-mm kernel compared against BSIFs. PVC was shown to have a large impact on the resultant IDIF for all scans with no-PVC IDIFs poorly matching the BSIFs. Figure 5 shows scatter plots of the correlation between AUC for PVC adjusted peaks (A), tails (B) and also the ratio between peaks and tails (C) for the optimal threshold, with an average AUC difference between peaks, tails and ratio of 5% ± 5.6%, 1% ± 11% and 6% ± 13%, respectively, with R^2^ values of 0.95, 0.70 and 0.76.

### Cerebral blood flow

**Fig. 6:**
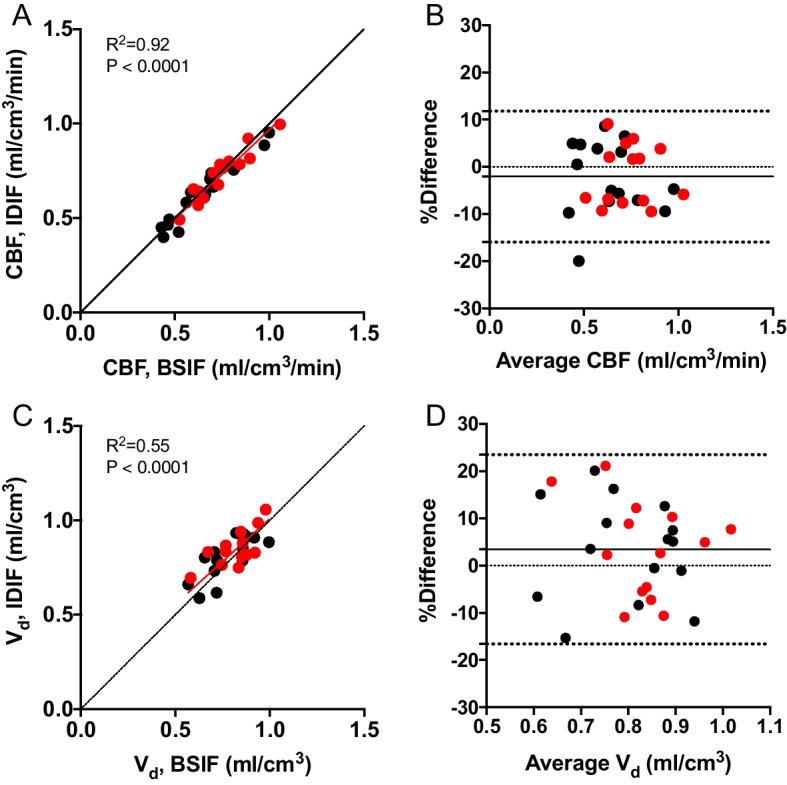
Scatter plots comparing whole-brain GM CBF (**A**) and *V*_d_ measurements (**C**) for BSIF and IDIF with baseline scans in black and post-acetazolamide scans in red. Bland–Altman plots are also shown for percentage differences for CBF (**B**) and *V*_d_ (**D**) against the average. GM: grey matter. *CBF* cerebral blood flow. *V*_d_ volume of distribution

Total GM CBF and *V*_d_ comparisons are shown in Fig. 6. The R^2^ between BSIF and IDIF CBF values was 0.92, the repeated measures can be seen in Additional file 1: Fig. S3 and showed an R^2^ of 0.915. GM CBF values typically showed good agreement with an average difference between the BSIF and IDIF CBF values of 2% ± 7% with a coefficient of variation (CoV) of 7.3% calculated as the standard deviation (SD) divided by the mean in the whole group (SD/mean). Results for whole-brain CBF values can be found in Additional file 1: Table S1 and Fig. S1.

**Fig. 7:**
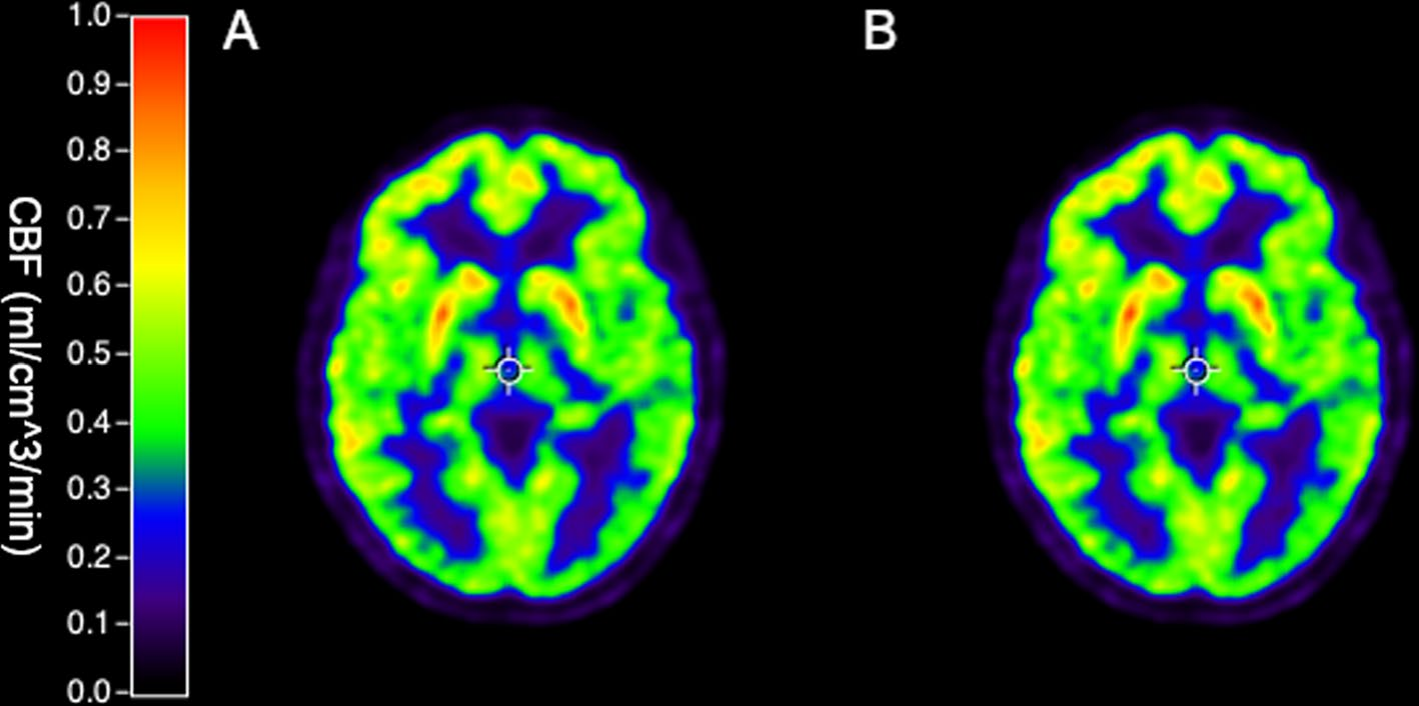
Parametric images of the CBF derived from IDIF after PVC (**A**) and BSIF derived CBF (**B**)

Within-subject correlation of BSIF- and IDIF-based rCBF values of the average of all VOIs in the Hammers atlas is reported in Table 1. The within-subject agreement was generally very high with an average R^2^ correlation of 0.99 and a minimum correlation of 0.966, as also visually illustrated in Fig. 7 which shows example CBF parametric images of the same representative subject seen in Fig. 4.

**Table 1:** Correlation and agreement between BSIF- and IDIF-based CBF values

Region	Variable	Baseline	Acetazolamide
Whole-brain GM CBF across subjects	Mean (SD) CBF (ml/cm^3^/min), BSIF	0.64 (0.18)	0.74 (0.14)
	Mean (SD) CBF (ml/cm^3^/min), IDIF	0.63 (0.16)	0.73 (0.14)
	Correlation (R^2^)	0.91	0.90
	Slope	0.92	0.96
	Bias	− 2.5%	− 1.7%
	Limits of agreement	− 17.8%	− 14.3%
		12.9%	10.9%
	SD of differences (ml/cm^3^/min)	0.047	0.047
	CoV of differences	7.3%	6.4%
rCBF within subjects	Mean (SD) correlation (R^2^)	0.99 (0.01)	0.99 (0.01)
	Mean (SD) slope	0.98 (0.16)	0.98 (0.20)

*BSIF* blood-sampled input function, *IDIF* image-derived input function, *CBF* cerebral blood flow

## Discussion

In this study, we propose a stand-alone, non-invasive method for deriving an input function from dynamic ^15^O–water PET scans to quantify CBF. Both the input functions themselves and the resultant CBF measures correlated well with the reference blood sample-derived metrics. AUC measurements showed good agreement between the IDIFs after PVC and BSIFs both in the average differences between AUC measurements and R^2^ correlations for the AUC comparisons. This translated into CBF and *V*_d_ measures where the CoV of the differences between IDIF- and BSIF-based baseline CBF values of 7.3%. This is lower than the CoV found in a previous test–retest study at 8.8% [43], and another study measuring a variation of 8.4% [44]. This indicates that differences between IDIF- and BSIF-based CBF values fall within the test–retest variability of CBF measurements using a BSIF, which can be seen as an indication that the presented IDIF method is quantitatively accurate and robust. The IDIF GM CBF and *V*_d_ values also showed a good overall correlation to the BSIFs as demonstrated in panels A and C in Fig. 6. Reactivity after acetazolamide administration in subjects was lower than previously seen in other studies, this result is likely due to an error in study design and subjects could have been administered a higher dose. However, this does not appear to be a true clinical finding in these patients.

Whilst the BSIF is often considered as the gold standard for the measurement of radiotracer concentration in plasma, it also contains measurement errors. Examples of this include the delay and dispersion correction, vascular structure, blood distribution and blood haematocrit which can lead to large errors when calculating metrics such as cerebral blood flow (CBF) in ^15^O–water PET scans [32]. Other ^15^O–water studies attempting to use IDIFs have used more complex methods of analysis such as machine learning-based approaches or the use of time-of-flight (TOF) MRI. These yielded R^2^ correlations for whole-brain GM CBF of 0.73–0.90 and an average bias of 3–18% [10, 12–15, 18], whilst our, PET-only method yielded an *R*^2^ correlation of 0.92 for GM CBF and an average bias of 2.1%. This suggests that there is a high level of reliability in our method with the additional benefit of not requiring complex analysis techniques or additional scans. CBF values were observed to be higher than those observed in previous studies, this could be explained by differences in the VOIs used where our study used a GM mask rather than a whole-brain mask leading to reductions in PVEs and increasing the CBF. Additionally, previous studies have used different models to estimate the CBF and which could lead to different values. Using a whole-brain mask gives values more aligned with those from previous studies (Additional file 1: Table S1). One possible explanation for the observed lower CBF in the whole brain compared to just the GM could be the presence of additional vasculature in white matter regions, which are included in the whole-brain mask but not in the GM mask. This additional vasculature could be contributing to the CBF values being lower due to the CBF in white matter regions reportedly being lower than in GM regions [45, 46]. Another possible explanation could be PVE, where there is an underestimation of the actual CBF values in the grey matter mask. The use of a whole-brain mask may reduce the impact of PVE, leading to the observed CBF values being higher.

Due to substantial PVE in the initial images, there was always a large difference in the BSIF and IDIF before PVC, with the IDIF values typically ~ 50% below the BSIF values. Alongside our carotid definition, we developed a method for accounting for the PVEs present in neurological PET images which attempts to account for the spill-in and spill-out. Despite our results being specific to ^15^O–water PET, it will be possible to also apply the technique to other tracers if the period of early tracer delivery is included in the scanning and the field-of-view includes the carotids. ^15^O–water has an advantage for the method development because it reaches equilibrium in tissue quickly and requires no metabolite corrections. It also has the advantage of comparatively short scan times, leading to fewer motion artefacts in the resultant images. For other tracers, there may therefore be additional requirements to make this technique feasible and this will be the subject of further studies.

### Limitations

Whilst we observed high reliability across subjects, some issues could affect the robustness of the presented methods. Firstly, delineation of the carotids for subjects with smaller than average carotid arteries as the resolution of the images is close to the diameter of a typical adult carotid artery (5–6 mm), this would also be the case for subjects with existing pathology that narrows or block the carotid arteries and could also lead to similar difficulties in resolving the carotids even at the highest resolution available. Additionally, patient motion may affect the accuracy of the results. Blood gas levels were not checked in these patients, due to this it is uncertain if reactivity changes were impacted by blood PaCO2 levels.

The method presented in this work requires high signal-to-noise first-pass images for the thresholding to perform correctly, and a high spatial resolution to enable accurate PVC. Both requirements are met by the use of block-sequential expectation maximisation, allowing full convergence whilst limiting image noise, including resolution recovery. Hence, a limitation of the current approach is that it cannot be readily applied to PET scanners with low resolution or less optimal reconstruction methods. Further research studies into the effects of different reconstructions should be conducted to establish how transferable our IDIF method is to images from other vendors.

An additional parameter that could be explored is the regularisation parameter β used during the penalised reconstruction. The effect of the β value on isolating the carotids or the subsequent IDIF was not investigated, this could have marginal but measurable improvements. Additionally, other reconstruction methods from other vendors have not been explored and so would need to be verified before implementation on a system without access to the specific implementation used in this work.

Due to the requirement for manual inspection of the PET data for each subject, the method for deriving the carotid masks and therefore the IDIFs is still somewhat time-consuming, and knowledge of the PET data is needed. Repeating the analysis from the beginning showed that the reproducibility of this method is high with a comparable R^2^ score on the second attempt. Current methods require manual inspection of the dynamic PET data to determine optimal VOI definition and temporal frame inclusion or exclusion. Whilst this method can be employed in other scenarios it requires specialistic knowledge and can suffer from the subjectivity of the reader in which frames to include or exclude. In this case, an automated method would allow the implementation without any need for specialist knowledge and would remove the subjectivity inherent in manual inspections. However, ad hoc analyses showed a substantially weaker correlation between the IDIFs and the BSIFs AUCs as well as subsequent GM CBF resulting from an automated approach (Additional file 1: Figs. S4 and S5) compared to using a manual approach (Figs. 5 and 6). Thus, although the encouraging result for the manual approach, further work is required for clinical routine ^15^O–water PET applications.

## Conclusion

This study shows that a robust IDIF can be produced based on images for dynamic ^15^O–water PET scans with no need for a corresponding MRI or complex analytical techniques. With further development of the method, adequate scanner resolution and robust reconstruction parameters, this technique can potentially be employed for clinical dynamic ^15^O–water PET scans and thereby making routine clinical use of quantitative CBF measurements with ^15^O–water PET feasible.

## Supplementary Information

**Additional file 1. Table S1**: Correlation and agreement between BSIF- and IDIF-based CBF values using a whole-brain mask. BSIF: Blood-sampled input function. IDIF: Image-derived input function. CBF: Cerebral blood flow. **Fig. S1**: GM mask used to derive CBF values (A), PET image (B) and fusion of mask and image (C). **Fig. S2**: Mean percentage bias of AUC of peaks (A) and tails (B) and the bias of peak-to-tail ratios (C) and GM CBF (D) for a range of Gaussian kernels and threshold levels using OSEM reconstruction algorithm. AUC: area under the curve. GM: grey matter. CBF: cerebral blood flow. OSEM: Ordered subset expectation maximisation. **Fig. S3**: CBF measurements comparing IDIF to BSIF when repeating the manually determined IDIFs. **Fig. S4**: Scatter plots comparing peak and tail (A) AUC of BSIF and IDIF, as well as peak-to-tail ratio (B). The black lines are lines of identity and the red lines are orthogonal regressions. **Fig. S5**: Scatter plots comparing whole-brain GM CBF (A) for BSIF and IDIF with baseline scans and post-acetazolamide scans. Bland–Altman plots are also shown for percentage differences for CBF (B) against the average. GM: grey matter. CBF: cerebral blood flow.

## Data Availability

The recorded MRI and PET images and information on the patients included in the study are not publicly available. Data derived from the images such as global cerebral blood flow values and image-derived input function time series are available upon request; however, a data-sharing agreement is needed. All software used in the study is available upon request without restriction.

## Abbreviations

AI: Artificial intelligence
AUC: Area under the curve
BSIFs: Blood-sampled input function
BSREM: Block sequential regularised expectation maximisation
CBF: Cerebral blood flow
CoV: Coefficient of variation
CT: Computed tomography
FoV: Field of view
GM: Grey matter
IDIF: Image-derived input function
MLEM: Maximum likelihood expectation maximisation
MRI: Magnetic resonance imaging
OSEM: Ordered subset expectation maximisation
PBIF: Population-based input function
PET: Positron emission tomography
PVC: Partial volume correction
PVE: Partial volume effect
SNR: Signal-to-noise ratio
TAC: Time activity curve
TOF: Time of flight
Vd: Volume of distribution
VOI: Volume of interest
