# Supplementary material for: Image-derived input functions from dynamic 15O–water PET scans using penalised reconstruction
Source: EJNMMI Phys. 2023 Mar 7;10:15. doi: 10.1186/s40658-023-00535-w (PMC9992469; doi:10.1186/s40658-023-00535-w)
Supplement: Supplementary file 1 — Additional file 1. Table S1: Correlation and agreement between BSIF- and IDIF-based CBF values using a whole-brain mask. BSIF: Blood-sampled input function. IDIF: Image-derived input function. CBF: Cerebral blood flow. Fig. S1: GM mask used to derive CBF values (A), PET image (B) and fusion of mask and image (C). Fig. S2: Mean percentage bias of AUC of peaks (A) and tails (B) and the bias of peak-to-tail ratios (C) and GM CBF (D) for a range of Gaussian kernels and threshold levels using OSEM reconstruction algorithm. AUC: area under the curve. GM: grey matter. CBF: cerebral blood flow. OSEM: Ordered subset expectation maximisation. Fig. S3: CBF measurements comparing IDIF to BSIF when repeating the manually determined IDIFs. Fig. S4: Scatter plots comparing peak and tail (A) AUC of BSIF and IDIF, as well as peak-to-tail ratio (B). The black lines are lines of identity and the red lines are orthogonal regressions. Fig. S5: Scatter plots comparing whole-brain GM CBF (A) for BSIF and IDIF with baseline scans and post-acetazolamide scans. Bland–Altman plots are also shown for percentage differences for CBF (B) against the average. GM: grey matter. CBF: cerebral blood flow. [file 40658_2023_535_MOESM1_ESM.docx]

Supplementary Material

| **Region** | **Variable** | **Overall** | **Baseline** | **Acetazolamide** |
| --- | --- | --- | --- | --- |
| Whole-brain CBF across subjects | Mean whole brain (SD) CBF  (ml/cm^3^/min), BSIF | 0.54 (0.11) | 0.42 (0.20) | 0.63 (0.12) |
|  | Mean whole brain (SD) CBF (ml/cm^3^/min), IDIF | 0.55 (0.14) | 0.44 (0.15) | 0.65 (0.13) |

**Supplementary Table 1** – Correlation and agreement between BSIF- and IDIF-based CBF values using a whole brain mask. BSIF: Blood-sampled input function. IDIF: Image-derived input function. CBF: Cerebral blood flow

B

A

C

**
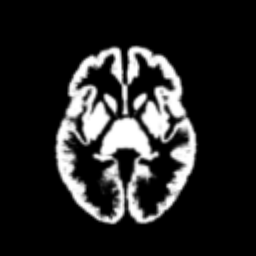

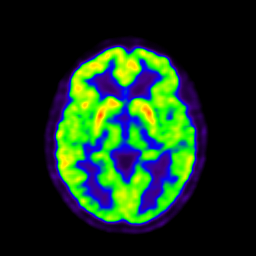

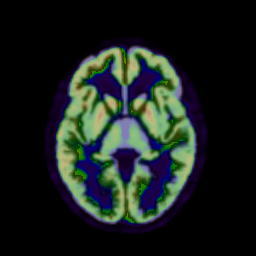
**

**Supplementary Figure 1.** – GM mask used to derive CBF values (A), PET image (B) and fusion of mask and image (C)

**Supplementary Methods**

Automation of the delineation of the carotids first involved a choice of frames that would include the carotids for all subjects. Frames 4-7 (corresponding to 20-40s) were selected and then averaged. Additionally, an ROI for all subjects was selected that would include the carotid for all subjects. The carotid masks were then created through a region growing method whereby the maximum pixel in the averaged image was used as an initial seed voxel, the region would be grown and then thresholded again at the chosen isocontour percentage and this would loop until the region did not grow any further.

These methods were employed in order to generate a more generalisable method for producing IDIFs, however, this method proved to be more susceptible to the differences between subject scans. For example, the frames in which the peak intensity was in the carotids and also the position of the subject’s head in the FOV leads to large variability in the resultant IDIFs. Below in Figure 1 are the results of the AUC comparisons and Figure 2 the CBF comparisons.

***Supplementary Figure 2.*** *Mean percentage bias of AUC of peaks (A) and tails (B) and the bias of peak-to-tail ratios (C) and GM CBF (D) for a range of gaussian kernels and threshold levels using OSEM reconstruction algorithm. AUC: area under the curve. GM: grey matter. CBF: cerebral blood flow. OSEM: Ordered subset expectation maximisation*

**Supplementary Figure 3.** CBF measurements comparing IDIF to BSIF when repeating the manually determined IDIFs.

**Supplementary Figure 4.** Scatter plots comparing peak and tail (A) AUC of BSIF and IDIF, as well as peak-to-tail ratio (B). The black lines are lines of identity and the red lines are orthogonal regressions.

**Supplementary Figure 5.** Scatter plots comparing whole-brain GM CBF (A) for BSIF and IDIF with baseline scans and post-acetazolamide scans. Bland-Altman plots are also shown for percentage differences for CBF (B) against the average. GM: grey matter. CBF: cerebral blood flow.
